# Relationships among heart rate variability, sleep disturbances, and fatigue in patients undergoing interventional therapy for hepatocellular carcinoma

**DOI:** 10.3389/fphys.2026.1923785

**Published:** 2026-10-07

**Authors:** Bo Xie, Yaqin Yun, Zhouhua Xu, Yue Meng, Mingxuan Peng, Yuqiao Xie, Huiyun Zhou, Hong Xu, Lingjie Xu

**Affiliations:** 1 Department of Interventional Medicine, The First Affiliated Hospital of Bengbu Medical University, Anhui, China; 2 School of Medical Imaging, Bengbu Medical University, Anhui, China; 3 School of Nursing, Bengbu Medical University, Anhui, China; 4 Faculty of Medicine, Macau University of Science and Technology, Macau, Macao SAR, China; 5 Department of Medical Oncology, The First Affiliated Hospital of Bengbu Medical University, Anhui, China

**Keywords:** autonomic nervous system, fatigue, heart rate variability, hepatocellular carcinoma, sleep disturbance

## Abstract

**Background:**

Fatigue and sleep disturbances are commonly experienced by patients with hepatocellular carcinoma (HCC). However, the potential association between these conditions and autonomic nervous system reflected by heart rate variability (HRV) is unclear. We aimed to evaluate the differences in HRV parameters among HCC patients with different degrees of fatigue and sleep disturbances, as well as the relationship between HRV parameters and fatigue and sleep disturbances.

**Methods:**

In this cross-sectional study, a total of 66 patients with HCC hospitalized for interventional therapy were enrolled. Patients completed questionnaires during hospitalization, including the Pittsburgh Sleep Quality Index (PSQI) and the Fatigue Scale-14 (FS-14), and underwent a 5-minute resting electrocardiogram (ECG) for HRV analysis. Group comparisons and logistic regression models adjusted for age were used to examine the associations between HRV parameters and symptom severity, with Benjamini-Hochberg false discovery rate (FDR) correction for multiple comparisons.

**Results:**

Acceleration capacity (AC) was significantly higher and low frequency (LF) was significantly lower in the high PSQI group compared to the low PSQI group (AC: p = 0.036; LF: p = 0.041); Compared to the low FS-14 group, the high FS-14 group had significantly higher mean heart rate (MeanHR) and AC, while most other HRV indices were significantly lower (MeanHR: p = 0.029; AC: p = 0.009). The results of the adjusted logistic regression model indicated that increased AC showed a marginal trend toward an association with poor sleep quality (p = 0.071), while MeanHR remained significantly associated with severe fatigue (OR = 1.098, p = 0.004). After FDR correction, the differences in eight HRV parameters between fatigue groups remained significant (all q < 0.05), whereas the differences between sleep-quality groups did not survive correction.

**Conclusions:**

Our findings suggest that alterations in HRV parameters, particularly mean heart rate (MeanHR) and acceleration capacity (AC), are associated with the severity of fatigue and sleep disturbances in HCC patients. Given the exploratory cross-sectional design, these metrics should be regarded as candidate non-invasive physiological correlates of autonomic dysfunction and symptom burden. Their clinical relevance requires confirmation in larger prospective studies.

## 1 Introduction

Hepatocellular Carcinoma (HCC) is a malignant tumor with a high incidence and mortality rate worldwide (Sung et al., 2021). Although minimally invasive interventional therapies such as transarterial chemoembolization (TACE) and radiofrequency ablation (RFA) have significantly improved patient survival outcomes, treatment-related debilitating symptoms remain prevalent. Fatigue is one of the most common, distressing, and frequently overlooked side effects of adult cancer and cancer treatment, and it may persist for years even after treatment completion (Al Maqbali et al., 2021). Additionally, cancer patients also commonly experience sleep disturbances. Studies indicate that approximately 60.7% of cancer patients have insomnia symptoms (Al Maqbali et al., 2022) and they often complain of difficulty falling asleep and insufficient sleep. These symptoms have become a critical clinical problem affecting prognosis and quality of life (QoL) through mechanisms such as reduced treatment adherence and exacerbated immune suppression (Bower, 2007; Ma et al., 2020).

Currently, the Pittsburgh Sleep Quality Index (PSQI) and Fatigue Scale-14 (FS-14) are widely employed in oncology due to their proven reliability and validity (Buysse et al., 1989; Chalder et al., 1993). However, as retrospective self-report measures, they are inherently limited by recall bias and subjective interpretation, which hinders objective symptom monitoring in routine clinical practice. This underscores the need to explore objective physiological correlates that may complement subjective assessments of fatigue and sleep disturbances.

The autonomic nervous system (ANS), a core regulatory component of the neuro-endocrine-immune network, is essential for maintaining body homeostasis. Increasing evidence suggests that HCC, typically arising from a background of chronic inflammation (Ringelhan et al., 2018), and its interventional treatments, which induce tumor necrosis and acute cytokine release (often manifesting as post-embolization syndrome), can significantly disturb body homeostasis. This elevated systemic inflammatory burden may activate the hypothalamic-pituitary-adrenal (HPA) axis and the sympathetic nervous system, ultimately manifesting as autonomic dysfunction (Bortolato et al., 2017; García-González et al., 2025). Consistent with this framework, autonomic dysfunction is well documented in chronic liver disease: HRV is reduced in patients with cirrhosis in parallel with systemic inflammation and neuropsychiatric impairment (Mani et al., 2009), and cardiac electrophysiological abnormalities are frequently observed even in the absence of overt heart disease (Zambruni et al., 2006). This dysfunction may manifest as abnormalities in heart rate variability (HRV). HRV serves as a non-invasive biomarker for evaluating ANS function, reflecting the balance between the sympathetic and parasympathetic nervous systems and cardiovascular regulatory capacity (Arakaki et al., 2023; Grégoire et al., 2023). Common linear analysis methods for HRV include time domain analysis and frequency-domain analysis (Shaffer and Ginsberg, 2017). Time domain analysis statistically characterizes successive heartbeat intervals. Key indicators include the mean heart rate (MeanHR), the standard deviation of normal RR intervals (SDNN) reflecting overall HRV and autonomic nervous system balance, and the root mean square of successive RR interval differences (RMSSD) primarily indicating parasympathetic activity (Cheng et al., 2022). Frequency domain analysis uses Fourier transforms to examine the distribution of heart rate time series across different frequency bands. The key indicators include: low frequency (LF, 0.04-0.15 Hz) and high frequency (HF, 0.15-0.4 Hz). While HF is widely accepted as a marker of parasympathetic activity, the physiological origin of LF is more complex, reflecting a mix of sympathetic and vagal influences, as well as baroreflex activity (Reyes Del Paso et al., 2013; Ishaque et al., 2021). Similarly, although the LF/HF ratio has traditionally been used to assess sympatho-vagal balance, recent evidence suggests this metric may be an oversimplification of autonomic interactions and should be interpreted with caution (Billman, 2013). Overall, linear HRV analysis serves as a valuable quantitative tool for assessing autonomic nervous system modulation.

Previous research in oncology has established a robust link between autonomic dysfunction and symptom burden. For instance, Fagundes et al. reported that breast cancer survivors with higher fatigue levels exhibited significantly lower HRV and elevated norepinephrine levels, suggesting a physiological shift toward autonomic dysregulation and parasympathetic withdrawal (Fagundes et al., 2011). Similarly, lower resting HRV has been associated with greater fatigue severity in cancer survivors, highlighting the potential of HRV as an objective biomarker for cancer-related symptoms (Crosswell et al., 2014).

However, existing research has mainly focused on breast cancer, with limited investigation into sleep disturbances and fatigue levels in patients undergoing interventional therapy for HCC. Furthermore, the relationship between these symptoms and autonomic dysfunction remains unexplored. Therefore, this study aims to explore the differences in HRV characteristics of HCC patients with different degrees of fatigue and sleep disturbances, as well as to clarify the relationship between fatigue, sleep disturbances, and HRV parameters. By clarifying these relationships, this exploratory study aims to generate hypotheses regarding autonomic correlates of symptom burden in this understudied population and to inform the design of future prospective studies.

## 2 Materials and methods

### 2.1 Subjects

This cross-sectional study was conducted between June 2024 and August 2024. A total of 66 HCC patients admitted to the Department of Interventional Radiology, the First Affiliated Hospital of Bengbu Medical University were enrolled using consecutive sampling. As this study was exploratory and hypothesis-generating in nature, no formal *a priori* sample size calculation was performed. All eligible patients admitted during the recruitment period were enrolled consecutively. This study serves as a preliminary pilot to identify clinical trends and provide effect size estimates for powering future large-scale prospective studies. During the study period, 78 hospitalized patients with HCC were assessed for eligibility, of whom 66 with complete questionnaire and ECG data were included in the final analysis and 12 were excluded.

The trial was conducted in strict accordance with the ethical standards outlined in the Declaration of Helsinki (1964) and its subsequent amendments. The study protocol received approval from the Ethics Review Committee of the First Affiliated Hospital of Bengbu Medical College. All patients provided written informed consent after understanding the study procedures. The inclusion criteria were as follows: (1) diagnosed with hepatocellular carcinoma after pathological histological examination; (2) no history of cardiovascular disease, such as coronary heart disease, arrhythmia, or cardiomyopathy, as verified by medical history, inpatient medical records, and a standard 12-lead ECG at admission; (3) no hearing or communication impairments. Patients with a history of other psychiatric disorders or current heavy tobacco or alcohol consumption were excluded.

### 2.2 Data collection

Inpatients completed sleep quality and fatigue questionnaires. To mitigate the potential physiological effects of cognitive activity or movement, patients were required to rest in a supine position for at least 10 minutes. Following this stabilization period, a 5-minute resting electrocardiogram (ECG) was recorded in the supine position using a single-lead Micro-ECG recorder (Healink-R211B, Healink Ltd., Bengbu, China). The sampling rate of ECG recorder was set at 400 Hz, and the signal bandwidth was adjusted to range from 0.6 to 40 Hz. The V6 lead configuration was employed to ensure a high-amplitude R-wave and optimal signal-to-noise ratio, which is critical for the accuracy of the QRS detection algorithm (Pan and Tompkins, 1985; Kligfield et al., 2007). The temperature in the ECG acquisition room was maintained within a comfortable range for human body (23 ± 1 °C). Patients were instructed to remain quiet and breathe regularly and steadily during the ECG measurement. All assessments were performed during the index hospitalization in which patients received image-guided interventional therapy for HCC, including transarterial chemoembolization (TACE), radiofrequency ablation (RFA), or other transarterial or ablative procedures; some patients underwent repeated treatment sessions. The timing of questionnaire administration and ECG recording relative to the interventional procedure was not standardized, which is acknowledged as a limitation. Pain intensity during hospitalization was assessed using a 0–10 visual analog scale (VAS). Respiration was not continuously monitored or paced during the recording.

### 2.3 Measurements

The Pittsburgh Sleep Quality Index: The PSQI assesses sleep quality over the past month (Buysse et al., 1989). The Chinese version, validated by Liu et al., demonstrates high reliability and validity (Liu, 1996). It consists of 19 items across seven components, with a global score ranging from 0 to 21. In this study, a total score greater than 7 was defined as indicating poor sleep quality.

Fatigue Scale‐14: The Fatigue Scale-14 (FS-14) was chosen to differentiate between physical and mental fatigue (Chalder et al., 1993). This 14-item scale comprises two dimensions: physical fatigue (items 1-8) and mental fatigue (items 9-14). The total score ranges from 0 to 14, with higher scores indicating greater severity. A score ≥ 6 was defined as severe fatigue (Cella and Chalder, 2010).

Heart rate variability analysis: The Pan-Tompkins algorithm was used to extract R-R interval (RRI) time series (Pan and Tompkins, 1985). Artifacts and ectopic beats were automatically corrected using the time-varying threshold and beat classification algorithm integrated into the Kubios HRV Premium software, as validated by Lipponen et al. (Lipponen and Tarvainen, 2019). HRV analysis was then performed to obtain a total of 9 HRV parameters. Across the 66 recordings, the median proportion of beats requiring artifact correction was 0.26% (range 0-8.75%); only one recording (1.5%) exceeded 5%, and the median proportion of analyzable signal was 100% (range 95.3-100%). No recording in the final cohort was excluded because of excessive artifacts.

The HRV linear time-domain analysis includes SDNN and RMSSD. The R-R interval time series was resampled at an interpolation rate of 4 Hz. Fast Fourier Transform was subsequently applied to obtain VLF, LF, HF, and LF/HF ratios. Furthermore, this study incorporated two additional metrics: Deceleration Capacity (DC) and Acceleration Capacity (AC). These measures quantify HRV related to deceleration and acceleration phases, respectively. While DC is a well-established marker of vagal tone, recent evidence suggests that at conventional scales (T = 1), AC reflects heart rate acceleration dynamics significantly influenced by vagal withdrawal, rather than pure sympathetic activation (Campana et al., 2010; Schneider et al., 2010; Pan et al., 2016). Thus, these metrics provide an integrated assessment of the heart’s capacity to decelerate and accelerate.

The above analysis was performed with Kubios HRV Premium software (version 4.0.2, Kubios Oy, Kuopio, Finland).

### 2.4 Statistical analysis

Statistical analyses were performed using SPSS 26.0 software. The normality of continuous variables was assessed using the Shapiro-Wilk test. Continuous variables with a normal distribution are expressed as mean ± standard deviation (SD), and comparisons between two groups were performed using the Student’s t-test. Variables with a non-normal distribution are presented as median [interquartile range (IQR)] and were analyzed using the Mann-Whitney U test. Categorical variables are expressed as frequencies and percentages (%), and differences were analyzed using the Chi-square test or Fisher’s exact test. To examine factors independently associated with symptom severity while preventing overfitting, multivariate logistic regression was performed in strict adherence to the “events per variable” (EPV) rule. Separate models were constructed for each HRV parameter to assess its independent value. Models were estimated as unadjusted (Model 1) and adjusted exclusively for Age (Model 2). P < 0.05 was considered significant. Because each adjusted model contained only one HRV parameter together with age, multicollinearity was evaluated by the correlation between the two covariates (all |r| < 0.30). Model calibration was assessed using the Hosmer-Lemeshow goodness-of-fit test. To account for multiple comparisons across the nine HRV parameters, the Benjamini-Hochberg procedure was applied within each outcome, and FDR-adjusted q values are reported alongside nominal P values. As complementary analyses, Spearman’s rank correlation coefficients were calculated between HRV parameters and continuous PSQI and FS-14 scores. A *post-hoc* power analysis indicated that, with 33 participants per fatigue group, the study had 80% power (two-sided alpha = 0.05) to detect a standardized between-group difference of approximately d = 0.70 (a medium-to-large effect); smaller effects may not have been detectable. Model discrimination was evaluated using the area under the receiver operating characteristic curve (AUC) with bootstrap 95% confidence intervals (2,000 resamples), and effect sizes for group comparisons are reported as rank-biserial correlations (r). Effect sizes for between-group comparisons are reported as rank-biserial correlations. Discrimination of the significant regression model was evaluated using the area under the receiver operating characteristic curve (AUC) with a bootstrap 95% confidence interval. As sensitivity analyses, the association between MeanHR and severe fatigue was re-examined after additional adjustment for hypertension and diabetes, and again after excluding patients with either condition. For the principal group comparisons, Hodges-Lehmann median differences are reported with 95% bootstrap confidence intervals (10,000 resamples).

## 3 Results

### 3.1 Baseline characteristics

This study enrolled 66 HCC patients undergoing interventional therapy. Baseline data showed that the average age of the patients was (59.2 ± 10.2) years, with 84.8% being male. The mean BMI was (22.8 ± 3.2) kg/m². Patients with hypertension and diabetes accounted for 33.3% and 16.7% respectively. The overall scores for the PSQI, FS-14 and all HRV indices are shown in **Table 1**. Seven patients (10.6%) reported a history of smoking and four (6.1%) reported a history of alcohol use. The median VAS pain score was 2.0 (IQR 0-3), and 16 patients (24.2%) reported moderate-to-severe pain (VAS ≥ 4).

**Table 1:** Demographical, clinical, questionnaire score and HRV measures of patients.

Variables	Values
Demographics	
N (female/male)	66 (10/56)
Age (years)	59.2 ± 10.2
BMI (kg/m^2^)	22.8 ± 3.2
Medical	
History of Diabetes (yes/no)	11/55
History of Hypertension (yes/no)	22/44
Smoking history (yes/no)	7/59
Alcohol history (yes/no)	4/62
Questionnaire	
PSQI	6.5 (5.0, 11.0)
FS-14	5.7 ± 2.8
Pain (VAS)	2.0 (0.0, 3.0)
HRV	
MeanHR	68.8 (64.1, 75.7)
SDNN	21.0 (12.8, 30.0)
RMSSD	12.2 (8.1, 17.7)
DC	8.4 (5.0, 14.2)
AC	-8.7 (-15.0, -5.1)
VLF	189.7 (71.4, 350.0)
LF	46.1 (15.6, 101.0)
HF	44.9 (19.9, 101.4)
LF/HF	1.4 (0.6, 2.2)

Values are expressed as mean (standard deviation) or median (inter-quartile range).

### 3.2 Differences in HRV indicators between different PSQI score groups

As shown in **Table 2**, the study divided the sample into two groups of participants. Among the 66 participants, 37 had good sleep quality (PSQI total score ≤ 7 points; age, 58.6 ± 11.2), accounting for 56.1% of the total sample. Twenty-nine patients had poor sleep quality (PSQI total score >7; age, 60.0 ± 8.9), accounting for 43.9% of the total sample. There were no statistically significant differences between the two groups in terms of age, gender ratio, BMI, hypertension, or diabetes. The low PSQI group had reduced autonomic nervous system complexity [AC: -9.3 (-17.6, -6.1) vs -6.0 (-11.4, -2.9), p = 0.036] and increased LF [72.8 (34.2, 115.8) vs 28.7 (13.9, 81.0), p = 0.041], However, neither of these differences remained statistically significant after Benjamini-Hochberg FDR correction across the nine HRV parameters (both q = 0.185); these unadjusted findings should therefore be regarded as exploratory. The corresponding effect sizes were small (AC: rank-biserial r = 0.303; LF: r = -0.295). The median differences between the poor and good sleep-quality groups (95% confidence interval) were 3.3 (0.2, 6.4) ms for AC and -29.2 (-55.4, -0.4) ms2/Hz for LF.

**Table 2:** Differences in HRV indicators between Low PSQI group and High PSQI group.

Variables	Low PSQI group (n=37)	High PSQI group (n=29)	*P*
Age	58.6 ± 11.2	60.0 ± 8.9	0.596
Sex			0.675
Female	5 (13.5%)	5 (17.2%)	
Male	32 (86.5%)	24 (82.8%)	
BMI	22.4 ± 2.8	23.4 ± 3.6	0.215
Diabetes			0.912
No	31 (83.8%)	24 (82.8%)	
Yes	6 (16.2%)	5 (17.2%)	
Hypertension			0.220
No	27 (73.0%)	17 (58.6%)	
Yes	10 (27.0%)	12 (41.4%)	
Smoking history			0.226
No	35 (94.6%)	24 (82.8%)	
Yes	2 (5.4%)	5 (17.2%)	
Alcohol history			0.312
No	36 (97.3%)	26 (89.7%)	
Yes	1 (2.7%)	3 (10.3%)	
MeanHR	68.7 (62.3, 74.1)	70.1 (64.7, 83.0)	0.281
SDNN	23.9 (17.3, 29.3)	18.4 (11.0, 31.2)	0.242
RMSSD	12.4 (9.1, 20.8)	11.2 (6.1, 15.8)	0.208
DC	9.3 (6.0, 15.7)	6.2 (3.3, 13.3)	0.062
AC	-9.3 (-17.6, -6.1)	-6.0 (-11.4, -2.9)	0.036
VLF	238.2 (88.0, 424.8)	143.1 (53.9, 309.2)	0.194
LF	72.8 (34.2, 115.8)	28.7 (13.9, 81.0)	0.041
HF	51.1 (29.9, 146.3)	36.5 (8.8, 76.3)	0.085
LF/HF	1.4 (0.6, 2.0)	1.5 (0.5, 2.6)	0.592

Low PSQI group: PSQI score ≤ 7; High PSQI group: PSQI score > 7; Data are presented as mean ± SD, median (IQR), or n (%). P-values derived from Mann-Whitney U test, t-test, or Chi-square test as appropriate. After Benjamini-Hochberg FDR correction across the nine HRV parameters, none of the between-group differences remained statistically significant (all q > 0.05).

### 3.3 Differences in HRV indicators between different FS-14 score groups

As shown in **Table 3**, 33 patients were assessed as having mild fatigue (FS-14 total score < 6 points; age, 55.9 ± 9.9), accounting for 50.0% of the total sample. There were 33 cases of severe fatigue (FS-14 total score ≥ 6 points; age, 62.6 ± 9.4), accounting for 50.0% of the total sample. The results indicated that the severe fatigue group had obvious autonomic nervous system regulation disorders. Patients of the severe fatigue group exhibited a significantly higher MeanHR(68.0 vs. 70.8 beats/min, p = 0.029) and AC(-10.4 vs. -6.0, p = 0.009). HRV indices were significantly reduced across multiple domains, including time domain [SDNN (25.1 vs. 17.2 ms, p = 0.015); RMSSD (14.4 vs. 9.7 ms, p = 0.030)]; DC (10.5 vs. 6.2, p = 0.041)], and frequency domain [VLF (250.6 vs. 99.5 ms2/Hz, p = 0.022); LF (76.9 vs. 28.7 ms2/Hz, p = 0.008); HF (58.9 vs. 31.5 ms2/Hz, p = 0.021)]. All of these differences remained significant after FDR correction (all q < 0.05), collectively suggesting broad autonomic dysregulation in patients with severe fatigue. Nevertheless, given the ongoing debate regarding the physiological interpretation of LF and related indices, these patterns should be interpreted cautiously as reflecting altered autonomic modulation rather than definitive sympathetic hyperactivation. The effect sizes for these differences were small to medium (rank-biserial |r| = 0.293-0.377). The corresponding effect sizes were moderate (rank-biserial r: MeanHR = 0.313; AC = 0.374; LF = -0.377; SDNN = -0.350; HF = -0.331; VLF = -0.328; RMSSD = -0.311; DC = -0.293). The median differences between the severe and mild fatigue groups (95% confidence interval) were 5.0 (0.6, 9.6) beats/min for MeanHR, -7.3 (-11.8, -1.7) ms for SDNN, -4.0 (-7.6, -0.3) ms for RMSSD, -3.4 (-6.6, -0.1) ms for DC, 4.1 (0.9, 7.4) ms for AC, -90.2 (-188.2, -13.8) ms2/Hz for VLF, -34.6 (-59.7, -10.9) ms2/Hz for LF, and -25.9 (-53.2, -4.2) ms2/Hz for HF.

**Table 3:** Differences in HRV indicators between Low FS-14 group and High FS-14 group.

Variables	Low FS-14 group (n=33)	High FS-14 group (n=33)	*P*
Age	55.9 ± 9.9	62.6 ± 9.4	0.007
Sex			0.170
Female	7 (21.2%)	3 (9.1%)	
Male	26 (78.8%)	30 (90.9%)	
BMI	23.1 ± 3.0	22.5 ± 3.4	0.428
Diabetes			0.741
No	27 (81.8%)	28 (84.8%)	
Yes	6 (18.2%)	5 (15.2%)	
Hypertension			1.000
No	22(66.7%)	22 (66.7%)	
Yes	11 (33.3%)	11 (33.3%)	
Smoking history			1.000
No	29 (87.9%)	30 (90.9%)	
Yes	4 (12.1%)	3 (9.1%)	
Alcohol history			0.613
No	30 (90.9%)	32 (97.0%)	
Yes	3 (9.1%)	1 (3.0%)	
MeanHR	68.0 (61.8, 72.1)	70.8 (66.5, 81.6)	0.029
SDNN	25.1 (19.6, 30.0)	17.2 (10.1, 31.0)	0.015
RMSSD	14.4 (10.2, 19.3)	9.7 (5.7, 16.2)	0.030
DC	10.5 (6.4, 16.7)	6.2 (3.4, 13.2)	0.041
AC	-10.4 (-19.2, -6.4)	-6.0 (-10.5, -3.1)	0.009
VLF	250.6 (117.1, 412.8)	99.5 (49.6, 286.9)	0.022
LF	76.9 (41.7, 118.1)	28.7 (11.1, 79.3)	0.008
HF	58.9 (31.6, 132.7)	31.5 (5.7, 69.7)	0.021
LF/HF	1.4 (0.5, 2.2)	1.3 (0.6, 2.2)	0.954

Low FS-14 group: FS-14 score < 6; High FS-14 group: FS-14 score ≥ 6; Data are presented as mean ± SD, median (IQR), or n (%). P-values derived from Mann-Whitney U test, t-test, or Chi-square test as appropriate. After Benjamini-Hochberg FDR correction across the nine HRV parameters, the differences in MeanHR, SDNN, RMSSD, DC, AC, VLF, LF, and HF remained statistically significant (all q < 0.05).

### 3.4 Associations between HRV parameters and symptom severity

**Table 4** presents the results of the logistic regression analyses. Separate models were constructed for each HRV parameter to assess its independent association with symptom severity. For severe fatigue, MeanHR remained significantly associated with severe fatigue after adjusting for age [OR (95% CI): 1.098 (1.031, 1.170), P = 0.004]. Acceleration Capacity (AC) showed a significant association with fatigue in the unadjusted model (P = 0.039) and maintained a marginal trend after adjustment (P = 0.093). For sleep quality, while no single parameter reached statistical significance (P < 0.05) in the adjusted models, MeanHR and AC exhibited borderline associations (P < 0.10). All models demonstrated adequate calibration (Hosmer-Lemeshow test, P > 0.05), and correlations between each HRV parameter and age were low (all |r| < 0.30), indicating no meaningful multicollinearity. Sensitivity analyses supported the robustness of the MeanHR-fatigue association, which remained significant after excluding patients with diabetes (OR = 1.098, 95% CI 1.024-1.176, P = 0.008), hypertension (OR = 1.097, 95% CI 1.019-1.182, P = 0.014), both conditions (OR = 1.094, 95% CI 1.010-1.184, P = 0.028), or a history of smoking or alcohol use (OR = 1.113, 95% CI 1.036-1.195, P = 0.003). The age-adjusted MeanHR model showed acceptable discrimination for severe fatigue (AUC = 0.785, 95% CI 0.660-0.884). The association remained essentially unchanged after additional adjustment for hypertension and diabetes (OR = 1.100, 95% CI 1.031-1.173, P = 0.004), and also persisted in a sensitivity analysis excluding patients with hypertension or diabetes (n = 37; OR = 1.094, 95% CI 1.010-1.184, P = 0.028), supporting the robustness of this finding against cardiovascular and metabolic comorbidities.

**Table 4:** Results from Logistic regression models.

Outcome	Predictor	Model 1		Model 2	
		OR (CI 95%)	*P*	OR (CI 95%)	*P*
PSQI	MeanHR	1.039 (0.990, 1.090)	0.120	1.043 (0.993, 1.097)	0.095
	SDNN	0.968 (0.925, 1.013)	0.161	0.969 (0.925, 1.014)	0.174
	RMSSD	0.983 (0.951, 1.017)	0.320	0.983 (0.951, 1.017)	0.320
	DC	0.944 (0.882, 1.011)	0.100	0.946 (0.884, 1.012)	0.108
	AC	1.072 (0.996, 1.155)	0.063	1.072 (0.994, 1.155)	0.071
	VLF	1.000 (0.998, 1.001)	0.496	1.000 (0.998, 1.001)	0.500
	LF	0.996 (0.989, 1.002)	0.203	0.996 (0.989, 1.003)	0.236
	HF	0.998 (0.993, 1.002)	0.277	0.998 (0.994, 1.002)	0.283
	LF/HF	1.206 (0.887, 1.638)	0.231	1.251 (0.909, 1.721)	0.170
FS-14	MeanHR	1.063 (1.007, 1.122)	0.026	1.098 (1.031, 1.170)	0.004
	SDNN	0.956 (0.912, 1.002)	0.059	0.957 (0.912, 1.005)	0.077
	RMSSD	0.994 (0.966, 1.024)	0.702	0.993 (0.963, 1.025)	0.676
	DC	0.973 (0.921, 1.029)	0.338	0.975 (0.923, 1.030)	0.366
	AC	1.079 (1.004, 1.161)	0.039	1.067 (0.989, 1.150)	0.093
	VLF	0.999 (0.997, 1.001)	0.199	0.999 (0.997, 1.001)	0.194
	LF	0.997 (0.992, 1.003)	0.384	0.999 (0.994, 1.005)	0.853
	HF	1.000 (0.998, 1.002)	0.755	1.000 (0.998, 1.002)	0.929
	LF/HF	1.098 (0.821, 1.470)	0.528	1.251 (0.912, 1.717)	0.165

OR, Odds Ratio; CI, Confidence Interval. Model 1 is an unadjusted model. Model 2 is adjusted for Age. All models demonstrated adequate calibration (Hosmer-Lemeshow test, P > 0.05). Sensitivity analyses excluding patients with diabetes, hypertension, or smoking/alcohol history yielded consistent estimates for the MeanHR-fatigue association (see text). The age-adjusted MeanHR model for severe fatigue showed acceptable discrimination (AUC = 0.785, 95% CI 0.660-0.884); the association persisted after additional adjustment for hypertension and diabetes (OR = 1.100, 95% CI 1.031-1.173) and after exclusion of patients with these conditions (OR = 1.094, 95% CI 1.010-1.184).

Complementary analyses using continuous symptom scores yielded consistent results. Higher continuous FS-14 scores were significantly correlated with higher AC (rho = 0.411, q = 0.005) and with lower SDNN, RMSSD, DC, VLF, LF, and HF (rho ranging from -0.282 to -0.381; all q < 0.05 after FDR correction), whereas MeanHR showed a borderline positive correlation (rho = 0.240, q = 0.059). In contrast, correlations between HRV parameters and continuous PSQI scores were weak and did not survive FDR correction (all q > 0.05). These results support the robustness of the association between autonomic indices and fatigue severity, while indicating that the association with sleep quality is weaker and exploratory. In addition, fatigue and sleep disturbance were moderately intercorrelated (Spearman rho = 0.510, P < 0.001), supporting their related but distinct symptom dimensions. In addition, PSQI and FS-14 total scores were moderately correlated (rho = 0.510, P < 0.001), consistent with the well-recognized interplay between sleep disturbance and fatigue.

## 4 Discussion

### 4.1 Principal findings

This study utilized the PSQI, FS-14 scales and HRV parameters to investigate the relationships among HRV, fatigue and sleep disorders in patients undergoing interventional therapy for hepatocellular carcinoma. Our results show that MeanHR was independently associated with the severity of fatigue after adjusting for age, and that differences in eight HRV parameters across fatigue groups remained significant after FDR correction. In addition, AC exhibited a borderline, hypothesis-generating association with sleep disturbances that was attenuated after adjustment for age and did not survive FDR correction. These findings indicate that HRV parameters are physiological correlates of symptom burden in this population and warrant confirmation in prospective studies.

### 4.2 Comparison with previous studies

While prolonging survival, interventional therapies are typically associated with substantial physiological and psychological burdens. The prevalence of fatigue and sleep disturbances among cancer patients ranges from 30% to 93.1%, depending on the diagnostic criteria used (Shahid et al., 2010; Chen et al., 2018; Al Maqbali et al., 2021). It is worth noting that fatigue and sleep disturbances often exhibit complex interactions. On one hand, chronic fatigue can lead to difficulty falling asleep and fragmented sleep. On the other hand, the decline of sleep quality may aggravate daytime fatigue, creating a vicious cycle. This not only limits patients’ normal daily activities but also profoundly impacts various aspects of their quality of life, including adherence to treatment and even the progression of cancer (Bower, 2007; Huang et al., 2021).

In addition, to assess the severity of fatigue and sleep disturbances, most studies rely on patient self-reports, using items such as “Overall, how would you rate your sleep quality?” (0 = very good; 3 = very poor). However, due to various factors, fatigue and sleep disturbances are often underestimated and inadequately treated (Ma et al., 2020). First, current primary assessment tools depend on patients’ subjective perceptions, lacking objectivity. Second, patients may gradually adapt to their impaired physical condition and perceive discomfort as normal. Third, although various pharmacological interventions for fatigue and sleep disturbances have been attempted in clinical practice, there remains a lack of evidence-based standard treatment protocols or clearly proven effective therapeutic approaches, and clinical guidelines have not yet established recommendations on this matter.

### 4.3 Potential mechanisms

Chronic sleep deprivation can alter the neuroendocrine system, making the body more susceptible to stress-related diseases such as cardiovascular disease and mental disorders (Zhao et al., 2025). In our unadjusted analysis, patients with poorer sleep quality exhibited elevated AC and significantly lower LF values. Considering the physiological basis of these parameters (Campana et al., 2010; Billman, 2013), this specific hemodynamic pattern likely reflects impairment in autonomic regulatory capacity. The suppressed LF indicates a reduction in overall autonomic modulation (or baroreflex sensitivity), while the elevated AC suggests a withdrawal of protective vagal activity, collectively pointing to autonomic dysfunction rather than simple sympathetic hyperactivation. Previous studies have also found that sleep disorders in glioma patients are associated with decreased HRV parameters reflecting changes in parasympathetic nervous system activity (Kitselaar et al., 2022), and our unadjusted findings initially aligned with this direction. However, it is well-established that autonomic function naturally declines with age, making age a potential confounder. After strictly adjusting for age to prevent overfitting, the association between AC and sleep quality was attenuated, remaining only marginally significant (P = 0.071). While this did not reach the traditional threshold for statistical significance, which was likely limited by the statistical power of our relatively small sample size, this trend raises the hypothesis that altered heart rate acceleration dynamics may be linked to sleep disturbance in HCC, consistent with patterns reported in other cancer populations, but this requires confirmation in adequately powered prospective studies. Furthermore, to rule out the potential confounding effect of centrally-acting hypnotics, an analysis of the PSQI sub-components revealed that the use of sleep medication was extremely low in our cohort (only 3 out of 66 patients, 4.5%), with no significant difference between the sleep-quality groups (P = 0.159). The vast majority of patients with high PSQI scores (89.7%) were medication-free, suggesting that hypnotic use is unlikely to fully account for the observed differences, although residual pharmacological confounding cannot be excluded. By quantifying the dynamics of inter-beat intervals independent of signal non-stationarities, PRSA-based parameters (AC and DC) are exceptionally robust against artifacts commonly found in short-term clinical recordings. While not necessarily superior to non-linear complexity metrics (e.g., entropy), AC and DC provide complementary value by capturing directional heart rate capacities that traditional time-domain metrics like SDNN may not effectively identify (Bauer et al., 2006).

This study observed significantly reduced values of SDNN, RMSSD, HF, and DC in patients with severe fatigue, indicating diminished parasympathetic tone. This finding is consistent with previous research results, which indicate that fatigue is closely related to ANS dysfunction (Fagundes et al., 2011). In addition, increased sympathetic nerve activity and decreased parasympathetic nerve activity can further activate the pro-inflammatory cytokine network. For example, Bower et al. found that compared to non-fatigued breast cancer survivors, breast cancer survivors with fatigue symptoms had significantly elevated levels of circulating pro-inflammatory cytokines (Bower, 2007). The observed alterations in sympathetic and parasympathetic activity were associated with cancer-related fatigue. Because this study was cross-sectional, it cannot determine whether autonomic alterations precede fatigue, result from fatigue, or reflect shared underlying factors. Although MeanHR is a preliminary metric lacking the refinement of beat-to-beat variability analysis, its independent association with severe fatigue is noteworthy. In HCC patients undergoing interventional therapy, an elevated resting MeanHR may reflect increased physiological stress in this population. Importantly, this association is unlikely to be specific to fatigue: elevated resting heart rate can also arise from systemic inflammation, post-embolization reactions, pain, anxiety, anemia, dehydration, infection, or overall disease severity, none of which were fully accounted for in the present analysis. MeanHR should therefore be interpreted as a non-specific physiological correlate rather than a specific marker of fatigue.

### 4.4 Clinical implications

In recent years, HRV parameters have increasingly been explored as non-invasive physiological correlates of symptom severity. Biofeedback interventions targeting HRV have been shown to be effective in improving sleep quality and alleviating fatigue in cancer populations. In particular, Lee et al. demonstrated that qigong and mindfulness interventions, using HRV changes as a key physiological indicator, can significantly enhance overall physical and mental health, strengthen emotional self-regulation, improve quality of life, effectively reduce fatigue, and improve autonomic nervous system dysfunction within a short period (Lee et al., 2023). Similarly, in Larkey’s study, psychosomatic interventions (such as qigong or Tai Chi) mainly caused significant decreases in HRV parameters related to sympathetic nerve activity, while psychosocial interventions (such as storytelling) were more likely to increase HRV indicators reflecting vagal tone (Larkey et al., 2020). Collectively, these studies indicate that interventions focusing on improving HRV are significant within comprehensive strategies for cancer patients, optimizing key clinical outcomes and informing clinical practice.

### 4.5 Strengths and limitations

Although this study provides novel insights, several limitations exist. First, the cross-sectional design precludes causal and temporal inference: autonomic alterations may contribute to, result from, or share common determinants with fatigue and poor sleep, and the modest sample size (n = 66) provided adequate power only for medium-to-large effects, rendering our findings hypothesis-generating rather than confirmatory. Second, the timing of questionnaire administration and ECG recording relative to the interventional procedure was not standardized, and post-procedural pain, fever, inflammatory responses, and medications may have simultaneously influenced HRV, fatigue, and sleep. Third, although cardiovascular disease was excluded by history, medical records, and 12-lead ECG, asymptomatic structural heart disease and paroxysmal arrhythmia cannot be fully ruled out; moreover, hypertension (33.3%), diabetes (16.7%), and the potential use of cardioactive medications (e.g., beta-blockers) may have independently affected resting heart rate and HRV and could not be fully accounted for. Fourth, several clinically important variables-including BCLC stage, Child-Pugh classification, liver function tests, hepatitis status, tumor burden, type and number of interventional treatment sessions, inflammatory markers, and the use of analgesics, sedatives, antidepressants, and corticosteroids-were not systematically collected, precluding adjustment for these potential confounders; residual confounding is therefore likely. Fifth, the PSQI assesses sleep over the preceding month, whereas HRV was derived from a single 5-minute awake recording, and time of day, fasting status, caffeine intake, and sleep on the preceding night were not standardized; the recorded HRV therefore cannot be assumed to reflect autonomic activity during sleep. Sixth, anxiety and depression, which may influence both sleep and HRV, were not assessed, and normal HRV should not be used to dismiss clinically meaningful subjective sleep complaints. Finally, while 5-minute recordings are clinically practical, they may lack the sensitivity of 24-hour Holter monitoring.

### 4.6 Future research

Future studies should employ larger, multicenter, prospective longitudinal designs with standardized measurement windows relative to interventional treatment, comprehensive clinical characterization (including liver function grading and tumor staging), systematic documentation of medications, and multimodal approaches combining HRV with inflammatory and metabolic biomarkers, in order to clarify the temporal and mechanistic relationships among autonomic function, sleep quality, and fatigue.

## 5 Conclusion

In conclusion, in this exploratory cross-sectional study of HCC patients undergoing interventional therapy, MeanHR was independently associated with severe fatigue, and AC showed a borderline association with sleep disturbances. These preliminary findings suggest an association between autonomic dysfunction and symptom burden in this population; however, they do not establish prediction, temporal sequence, or causality, and normal HRV values should not be used to dismiss clinically meaningful symptom complaints. These preliminary findings support an association between autonomic dysfunction and symptom burden in this population. Larger prospective multicenter studies are needed to clarify the temporal relationships and clinical relevance of these associations.

## Data Availability

The raw data supporting the conclusions of this article are not publicly available due to patient privacy concerns but are available from the corresponding author on reasonable request.
